# Depressive disorder and grief following spontaneous abortion

**DOI:** 10.1186/s12888-016-0812-y

**Published:** 2016-04-12

**Authors:** Susil Kulathilaka, Raveen Hanwella, Varuni A. de Silva

**Affiliations:** University Psychiatry Unit, National Hospital of Sri Lanka, Colombo, Sri Lanka; Faculty of Medicine, University of Colombo, Colombo, Sri Lanka

## Abstract

**Background:**

Abortion is associated with moderate to high risk of psychological problems such as depression, use of alcohol or marijuana, anxiety, depression and suicidal behaviours. The increased risk of depression after spontaneous abortion in Asian populations has not been clearly established. Only a few studies have explored the relationship between grief and depression after abortion.

**Methods:**

A study was conducted to assess the prevalence and risk factors of depressive disorder and complicated grief among women 6–10 weeks after spontaneous abortion and compare the risk of depression with pregnant women attending an antenatal clinic. Spontaneous abortion group consisted of women diagnosed with spontaneous abortion by a Consultant Obstetrician. Women with confirmed or suspected induced abortion were excluded. The comparison group consisted of randomly selected pregnant, females attending the antenatal clinics of the two hospitals. Diagnosis of depressive disorder was made according to ICD-10 clinical criteria based on a structured clinical interview. This assessment was conducted in both groups. The severity of depressive symptoms were assessed using the Patients Health Questionnaire (PHQ-9). Grief was assessed using the Perinatal Grief Scale which was administered to the women who had experienced spontaneous abortion.

**Results:**

The sample consisted of 137 women in each group. The spontaneous abortion group (mean age 30.39 years (SD = 6.38) were significantly older than the comparison group (mean age 28.79 years (SD = 6.26)). There were more females with ≥10 years of education in the spontaneous abortion group (*n* = 54; SD = 39.4) compared to the comparison group (*n* = 37; SD = 27.0). The prevalence of depression in the spontaneous abortion group was 18.6 % (95 CI, 11.51–25.77). The prevalence of depression in the comparison group was 9.5 % (95 CI, 4.52–14.46). Of the 64 women fulfilling criteria for grief, 17 (26.6 %) also fulfilled criteria for a depressive episode.

The relative risk of developing a depressive episode after spontaneous abortion was significantly higher than in females with a viable pregnancy (RR = 2.19, 95 % CI, 1.05 to 4.56). After adjustment for age and period of amenorrhoea, the difference was not significant. Prevalence of complicated grief was 54.74 % (95 % CI, 46.3–63.18).

**Conclusion:**

The relative risk of developing a depressive episode after spontaneous abortion was not significantly higher compared to pregnant women after taking into account age and period of amenorrhoea (POA). Almost half the women developed complicated grief after spontaneous abortion. Of these, a significant proportion also had features of depressive disorder.

**Electronic supplementary material:**

The online version of this article (doi:10.1186/s12888-016-0812-y) contains supplementary material, which is available to authorized users.

## Background

Spontaneous abortion is the involuntary termination of a nonviable intrauterine pregnancy before 28 weeks of gestation [1]. It is the commonest complication in pregnancy [2]. Spontaneous abortion is associated with moderate to high risk of psychological problems such as depression, use of alcohol or marijuana, anxiety and suicidal behaviours [3, 4].

Early pregnancy loss leads to symptoms of grief such as sadness, yearning, social isolation and guilt. [5]. A sense of loss is common while some women experience guilt and anger after spontaneous abortion [6]. These features are present even when the pregnancy was not planned [7]. The partner and other family members too can experience psychological distress [8]. Grief is a normal response to loss. In some, bereavement can result in complicated grief and depression. Longer-lasting psychological, social, and health status changes follow the initial depressive, but not the grief reactions. Depression after spontaneous abortion is often unrecognized by medical professionals [9].

Not all females experience adverse mental health consequences after spontaneous abortion. Women who have a past history of depression, women who are childless, have poor social support or pre-existing relationship difficulties are at risk for complicated grief and depression [8, 10, 11].

The natural course of psychological morbidity following spontaneous abortion is unclear. In the majority, grief decreases within 3–4 months but there is evidence that some females display symptoms of grief and depression one year later [3, 11, 12].

Most studies on the psychological impact of spontaneous abortion have been carried out in Western countries. Expression of grief and depression may show cultural variation [13]. Open expression of emotions such as sadness may not be considered appropriate in certain cultures and people may somatize their distress [14]. Therefore, there is a need for studies from non-Western cultures.

Most females experience grief after spontaneous abortion [3, 4]. Many who experience grief will recover naturally, but a proportion will develop clinical depression which requires treatment [3, 4]. Only a few studies have explored the relationship between grief and depression after spontaneous abortion.

Because of the small number of studies from non Western cultures and the need to explore the relationship between grief and depression after spontaneous abortion, we conducted a cohort study to assess the prevalence and risk factors of depressive disorder and intensity of grief among women after spontaneous abortion and compare the risk of depression with pregnant women attending an antenatal clinic. The study also explored views regarding the type of psychological care the patients required.

## Methods

This cohort study was conducted in the Obstetric and Gynaecology units of Teaching Hospital Anuradhapura in the North Central province and Base Hospital Avissawella in the Western Province of Sri Lanka. Spontaneous abortion was defined as the involuntary termination of a nonviable intrauterine pregnancy before 28 weeks of gestation. Women who presented with clinical features suggestive of spontaneous abortion were examined by a consultant obstetrician. An ultrasound scan was carried out in all patients. Based on these findings the diagnosis of spontaneous abortion was confirmed by the consultant obstetrician. Those who had a complete spontaneous abortion were invited to participate in the study. Women with a history suggestive of induced abortion were excluded. The comparison group consisted of randomly selected pregnant, females, with a period of amenorrhoea (POA) ≤28 weeks, who had not experienced any type of abortion in the previous 12 months, attending antenatal clinics of Teaching Hospital Anuradhapura and Base Hospital Avissawella. The sampling frame was all females with POA ≤28 weeks included in the antenatal register. The comparison group was selected by simple random sampling. The selected females were interviewed to find out if they had experienced a spontaneous abortion during the past 12 months.

The sample size was calculated to detect risk factors with an odds ratio of 2.0. The sample consisted of 137 each from spontaneous abortion and comparison groups. Women who fulfilled inclusion criteria were recruited consecutively until the desired sample size was achieved.

### Outcome measures

Data was collected 6–10 weeks after spontaneous abortion. A questionnaire was used to collect demographic data. Diagnosis of depressive disorder was made according to ICD-10 clinical criteria based on a structured clinical interview conducted by a consultant psychiatrist. The translated and validated version of the Patient Health Questionnaire (PHQ-9) was used to assess the severity of the depressive disorder [15]. PHQ-9 is a nine-item instrument that assesses symptoms of depression as listed in the DSM-IV. Each item is scored from 0 (not at all), to 3 (nearly every day). The total scores can range from 0 (no depressive symptoms) to 27 (all symptoms occurring daily). A total score of scores of 0 to 4 represents a minimal level of depression; 5 to 9, mild; 10 to 14, moderate; 15 to 19, moderately severe; and 20 to 27, severe. The PHQ-9 was completed by the patient and scored by the clinician.

Grief was assessed using the Perinatal Grief Scale (PGS) [16, 17]. The PGS was developed to assess the resolution of grief following spontaneous abortion, fetal or neonatal death, or ectopic pregnancy [17]. It consists of three subscales- “Active Grief”, “Difficulty Coping”, and “Despair”. The 33 item shorter version has good correlation (0.98) with the original 104 item questionnaire. The internal reliability coefficient (Cronbach’s α) ranges from .92 to .96 [17].

The 33 item short version of the PGS was translated to Sinhala. A combined qualitative and quantitative approach was used for the translation of the PGS [18]. A panel of three doctors who were bilingual individually translated the scale into Sinhala a language spoken by about 75 % of Sri Lankans. Final translation was selected by consensus among all three translators. A bilingual expert who was not familiar with the original scale back translated it to English. This English translation was compared with the original scale and necessary adjustments were made to prepare the final Sinhala Version. The translated scale was pretested on a group of 20 people in the community. Face, content and consensual validity of the Sinhala version was assessed by a panel of experts comprising consultant community physician, consultant psychiatrist, and psychiatric social worker.

Each item of the PGS was scored from 1 (strongly agree) to 5 (strongly disagree). The total score was arrived at by first reversing all the items except 11 and 33. The total score ranges from 33 to 165. A score of 90 or above was used as the cutoff point to identify the presence of grief [17].

The clinical interview to assess depression according to ICD-10 criteria was administered to both groups. The PGS was administered to the spontaneous abortion group.

### Data analysis

The difference between groups for continuous variables was assessed using t test. Categorical variables were assessed using the chi-square test. Relative risk and adjusted relative risk was calculated using the generalized linear models for the binomial family. Data was analysed using Stata version 12.0.

## Results

The sample consisted of 137 women who had experienced spontaneous abortion (spontaneous abortion group) and 137 women attending an antenatal clinic with a viable pregnancy (comparison group). The socio-demographic characteristics are shown in Table 1. There were no significant differences between the spontaneous abortion group and the comparison group in ethnicity, religion or income. The majority of participants were Sinhalese and Buddhist, reflecting the ethnic and religious distribution of the study areas. The spontaneous abortion group was significantly older than the comparison group. Fifty-four (39.4 %) females in the spontaneous abortion group had ≥10 years of education compared to 37 (27.0) in the comparison group. The period of amenorrhoea was less than 12 weeks in the majority of women with spontaneous abortion (*n* = 89; 71.8 %) compared to women in the comparison group (*n* = 33; 24.6).

**Table 1:** Demographic and clinical characteristics of spontaneous abortion group and comparison group

	Spontaneous abortion group (*N* = 137)	Comparison group (*N* = 137)	Significance
Age in years mean (SD)	30.39 (6.38)	28.79 (6.25)	*t* = 2.09 *p* = 0.037
Race			Chi 0.71; *df* = 2; *p* = 0.70
Sinhala	127 (93.4)	125 (91.2)	
Tamil	5 (3.7)	8 (5.8)	
Muslim	4 (2.9)	4 (2.9)	
Religion			*χ* ^2^ = 1.19; *df* = 3; *p* = 0.76
Buddhist	122 (89.1)	116 (84.7)	
Christian	7 (5.1)	10 (7.3)	
Hindu	4 (2.9)	6 (4.4)	
Islam	4 (2.9)	5 (3.6)	
Education			
10 years or less	83 (60.6)	100 (73.0)	*χ* ^2^ = 4.76; *df* = 1; *p* = 0.03
More than 10 years	54 (39.4)	37 (27.0)	
Employment			*χ* ^2^ = 0.50; *df* = 1; *p* = 0.48
Employed	32 (23.4)	27 (19.9)	
Unemployed or housewife	105 (76.6)	109 (80.1)	
Income			*χ* ^2^ = 2.84; *df* = 3; *P* = 0.42
Less than Rs. 10000	31 (22.6)	27 (19.7)	
Rs. 10000–15000	24 (17.5)	19 (13.9)	
Rs. 15000–20000	41 (29.9)	54 (39.4)	
>Rs. 20000	41 (29.9)	37 (27.0)	
Living children			*χ* ^2^ = 2.48; *df* = 1; *P* =0.12
Yes	80 (58.4)	67 (48.9)	
No	57 (41.6)	70 (51.1)	
Period of amenorrhoea			*χ* ^2^ = 63.8; *df* =2; *P* <0.001
≤12 weeks	93 (70.5)	33 (24.6)	
13–18 weeks	32 (24.2)	54 (40.3)	
19–28 weeks	7 (5.3)	47 (35.1)	
Unknown	5 (3.6)	3 (2.2)	

### Outcome measures

In the spontaneous abortion group 19 (13.87 %) refused to participate in the clinical interview to detect depression. Twenty-two females in the spontaneous abortion group and 13 females in the comparison group fulfilled ICD-10 criteria for a depressive episode. The prevalence of depression was 18.6 % (95 % CI, 11.51–25.77) in women after spontaneous abortion and 9.5 % (95 % CI, 4.52–14.46) in the comparison group. The relative risk of developing a depressive episode after spontaneous abortion was 1.96 (95 % CI, 1.04–3.73) compared to pregnancy. However when we adjusted for age and POA there was no significant increase in relative risk (adjusted RR 1.42 (0.65–3.07) *p* = 0.38).

According to the PHQ-9 moderate- severe depression was seen in 20 (16.9 %) in the spontaneous abortion group and 16 (11.8 %) in the comparison group (Table 2). Seventy-five women scored 90 or more in the Perinatal Grief Scale. Prevalence of complicated grief was 54.74 % (95 % CI, 46.3–63.18).

**Table 2:** Severity of depression according to PHQ-9

	Spontaneous abortion group *N* = 119	Comparison group *N* = 136
	Number (%)	Number (%)
Minimal	90 (75.6)	81 (59.6)
Mild	9 (7.6)	39 (28.7)
moderate	11 (9.2)	11 (8.1)
Moderately severe	6 (5.0)	5 (3.7)
Severe	3 (2.5)	0 (0)
Total	119	136

Table 3 shows the number of women who experienced grief and depression after spontaneous abortion. Forty-nine (41.5 %) had neither depression nor grief. Of the 64 women fulfilling criteria for grief, 17 (26.6 %) also fulfilled criteria for a depressive episode. Only 5 (9.3 %) women had depression without grief.

**Table 3:** Grief and depression in women after spontaneous abortion

	No grief *N* (%)	Grief positive	Total
Depression positive	5 (9.3)	17 (26.6)	22
Depression negative	49 (90.7)	47 (73.4)	96
Total	54	64	118

Only 21 (15.3 %) women thought that they were responsible for the abortion. Eighty-four (61.3 %) stated that they were not responsible for it while 32 (23.4 %) were unsure. The majority of women, 95 (69.3 %) felt that they needed to talk about the abortion. The preferred type of support is detailed in the Additional file 1: Table S1.

## Discussion

In this study, we found that 6–10 weeks after spontaneous abortion 18.6 % of females fulfilled criteria for depression and 54.2 % fulfilled criteria for complicated grief. Although the relative risk of depression was 1.96 (95 % CI, 1.04–3.73) when we adjusted for age and POA the difference was no longer significant. Comorbid depression was present in 26.6 % of women with grief.

Although unadjusted logistic regression analysis found significant difference in relative risk of depression the difference was no longer significant after we adjusted for age and POA. We adjusted for age and POA as they were significantly different between the two groups. It is possible that we have over-adjusted and age and POA do not affect the causal relationship but has reduced the precision [19]. Therefore the findings have to be interpreted with caution.

We found that the rate of depression after spontaneous abortion of 18.6 % was similar to that reported in other studies. Although in some Western populations the rate of depression after spontaneous abortion is in the range of 40–50 %, a post-1990 review of literature reported lower levels around 20 % [10, 20–22]. The rate of depression after spontaneous abortion among Asian women may be lower. This may be due to several factors such as better social support and a good relationship with the partner. In Chinese women in Hong Kong, the prevalence of depression 6 weeks after abortion was 12 % while another study reported prevalence of 10 % 3 months after spontaneous abortion [23, 24].

Socio-cultural factors influence the prevalence of depression in the general population. A population-based study in Sri Lanka reported life time depression prevalence of 6.6 % rising to 11.2 % if the functional impairment criterion (clinically significant distress or impairment in social occupational or other important areas of functioning) was excluded [25]. The prevalence of depression in our spontaneous abortion group was higher than that among females in the general population. Other studies from Sri Lanka show higher rates of depression in patients with chronic physical illness. Prevalence of depression was 27.9 % among patients with chronic renal failure in Sri Lanka and 37.9 % among patients with Parkinson’s disease [26, 27]. The prevalence of depression after spontaneous abortion in our sample was lower than that in patients with chronic medical conditions.

A systematic review of 21 published studies reported no increase in risk of depression after induced abortion while other reviews have reported increased risk of negative mental health consequences [28, 29]. Risk factors for depression after induced abortion are different to those after spontaneous abortion. The psychological sequelae of spontaneous abortion and induced abortion are different. A 5 year follow-up study found that women who had a miscarriage had significantly more grief and feelings of loss while feelings of guilt were significantly more in the induced abortion group [12]. The comparison groups used are also different. Most studies assessing the risk of depression after spontaneous abortion have used pregnant females as the comparison group while women with unwanted pregnancies have been used as a comparison group for assessing risk after induced abortion. Women with unwanted pregnancies may be at higher risk of depression because of financial and relationship issues and poor social support. This may explain why some studies have not found an increased risk of depression after induced abortion. The point in time when assessments are carried out also influence results as the prevalence of depression reduces with time. Immediately after miscarriage 26.8 % of patients scored high on the Beck Depression Inventory (>or = 12), which reduced to 18.4 % at 3 months, 16.4 % at 6 months, and 9.3 % at 1 year after miscarriage [30]. Therefore, the risk of depression compared to non pregnant controls also decreases with time [11, 30].

This study also explored the relationship between depression and grief. More than half the women fulfilled criteria for grief, of them 26.6 % had depression. According to DSM-IV diagnostic criteria diagnosis of major depressive disorder is permitted only if the symptoms are not better accounted for by bereavement [31]. Therefore, it is important to explore the overlap between the two conditions.

Our study shows that a sizable proportion of women after spontaneous abortion fulfill criteria for depressive disorder. DSM-5 allows the diagnosis of depressive disorder in those with features of bereavement. According to the DSM-5 response to bereavement may resemble a depressive episode. Although such symptoms may be understandable the diagnosis of a depressive episode is allowed if the individual fulfills the diagnostic criteria [31]. Symptoms of grief and depression diminish over time. Longitudinal studies have found that women who are more distressed initially continue to be distressed even 1 year after the event [30]. Our findings suggest that the group of women who experience both grief and depression requires medical attention.

This study had several strengths. It is one of the few studies which assessed depression and grief using standardized scales. The diagnosis of depression was confirmed following a structured clinical interview. The major weakness of the study was that data was collected only at a single point in time. The comparison group was not matched, therefore there were differences in age and POA, which we adjusted for in the statistical analysis.

## Conclusions

The findings of this study have several implications. Although unadjusted logistic regression analysis found significant difference in relative risk of depression the difference was no longer significant after we adjusted for age and POA. The majority of women said they would like to talk to a doctor about the abortion. Women with features of depression should be referred to mental health services for specialized assessment and care.

### Ethics approval and consent to participate

Ethical clearance was obtained from Ethics Review Committee of Colombo South Teaching Hospital. Since the Base Hospital Avissawella did not have an Ethics Review Committee we obtained ethical clearance from the Ethics Review Committee of Colombo South Teaching Hospital which is a member of the Forum of Ethics Review Committees of Sri Lanka and provides ethical clearance for studies conducted in institutions with no ethics committees. Written informed consent was obtained after nature and the possible consequences of the study were explained to the participants. Participants who were diagnosed as having depressive disorder were referred to the psychiatry unit for further assessment and treatment.

### Consent for publication

Not applicable.

### Availability of data and materials

Data will be shared upon request.

## Additional file

Additional file 1: Table S1. Type of support preferred by the females after spontaneous abortion. (DOCX 15 kb)

## Abbreviations

CI: confidence interval
DSM-IV: diagnostic and statistical manual-IV
ICD-10: International Classification of Mental and Behavioural Disorders
PGS: perinatal grief scale
PHQ-9: patient health questionnaire
POA: period of amenorrhoea
RR: risk ratio
SD: standard deviation

## References

[1] Neugebauer R, Kline J, Shrout P, Skodol A, O’Connor P, Geller PA (1997). Major depressive disorder in the 6 months after miscarriage. JAMA.

[2] Nybo Andersen AM, Wohlfahrt J, Christens P, Olsen J, Melbye M (2000). Maternal age and fetal loss: population based register linkage study. BMJ.

[3] Coleman PK (2011). Abortion and mental health: quantitative synthesis and analysis of research published 1995–2009. Br J Psychiatry.

[4] Bellieni CV, Buonocore G (2013). Abortion and subsequent mental health: Review of the literature. Psychiatry Clin Neurosci.

[5] Iles S (1989). The loss of early pregnancy. Baillieres Clin Obstet Gynaecol.

[6] Frost M, Condon JT (1996). The psychological sequelae of miscarriage: a critical review of the literature. Austr N Z J Psychiatry.

[7] Zaccardi R, Abbott J, Koziol-McLain J (1993). Loss and grief reactions after spontaneous miscarriage in the emergency department. Ann Emerg Med.

[8] Kersting A, Wagner B (2012). Complicated grief after perinatal loss. Dialogues Clin Neurosci.

[9] Friedman T, Gath D (1989). The psychiatric consequences of spontaneous abortion. Br J Psychiatry.

[10] Neugebauer R, Kline J, O’Connor P, Shrout P, Johnson J, Skodol A, Wicks J, Susser M. Depressive symptoms in women in the six months after miscarriage. Am J Obstet Gynecol. 1992;166(1 Pt 1):104–9. 10.1016/0002-9378(92)91839-3 1733177

[11] Janssen HJ, Cuisinier MC, de Graauw KP, Hoogduin KA (1997). A prospective study of risk factors predicting grief intensity following pregnancy loss. Arch Gen Psychiatry.

[12] Broen AN, Moum T, Bodtker AS, Ekeberg O (2005). The course of mental health after miscarriage and induced abortion: a longitudinal, five-year follow-up study. BMC Med.

[13] Kirmayer LJ (2001). Cultural variations in the clinical presentation of depression and anxiety: implications for diagnosis and treatment. J Clin Psychiatry.

[14] Kirmayer LJ, Robbins JM, Dworkind M, Yaffe MJ (1993). Somatization and the recognition of depression and anxiety in primary care. Am J Psychiatry.

[15] Hanwella R, Ekanayake S, de Silva VA (2014). The Validity and Reliability of the Sinhala Translation of the Patient Health Questionnaire (PHQ-9) and PHQ-2 screener. Depress Res Treat.

[16] Zisook S, Devaul RA, Click MA (1982). Measuring symptoms of grief and bereavement. Am J Psychiatry.

[17] Toedter LJ, Lasker JN, Janssen HJ (2001). International comparison of studies using the perinatal grief scale: a decade of research on pregnancy loss. Death Stud.

[18] Sumathipala AMJ (2000). New approach to translating instruments for cross-cultural research: a combined qualitative and quantitative approach for translation and consensus generation. Int J Methods Psychiatr Res.

[19] Schistermana EF, Coleb SR, Plattc RW (2009). Overadjustment bias and unnecessary adjustment in epidemiologic studies. Epidemiology.

[20] Simon NM, Rothman D, Goff JT, Senturia AG (1969). Psychological factors related to spontaneous and therapeutic abortion. Am J Obstet Gynecol.

[21] Neugebauer R, Kline J, O’Connor P, Shrout P, Johnson J, Skodol A, Wicks J, Susser M. Determinants of depressive symptoms in the early weeks after miscarriage. Am J Public Health. 1992;82(10):1332–9. 10.2105/ajph.82.10.1332 PMC1695859 1415855

[22] Bradshaw Z, Slade P (2003). The effects of induced abortion on emotional experiences and relationships: a critical review of the literature. Clin Psychol Rev.

[23] Lee DT, Wong CK, Cheung LP, Leung HC, Haines CJ, Chung TK (1997). Psychiatric morbidity following miscarriage: a prevalence study of Chinese women in Hong Kong. J Affect Disord.

[24] Sham A, Yiu M, Ho W (2010). Psychiatric morbidity following miscarriage in Hong Kong. Gen Hosp Psychiatry.

[25] Ball HA, Siribaddana SH, Kovas Y, Glozier N, McGuffin P, Sumathipala A, Hotopf M. Epidemiology and symptomatology of depression in Sri Lanka: a cross-sectional population-based survey in Colombo District. J Affect Disord. 2010;123(1–3):188–96. 10.1016/j.jad.2009.08.014 PMC2946561 19762085

[26] Sumanathissa M, De Silva VA, Hanwella R (2011). Prevalence of major depressive episode among patients with pre-dialysis chronic kidney disease. Int J Psychiatry Med.

[27] Ketharanathan T, Hanwella R, Weerasundera R, de Silva VA (2014). Major depressive disorder in Parkinson’s disease: a cross-sectional study from Sri Lanka. BMC Psychiatry.

[28] Charles VE, Polis CB, Sridhara SK, Blum RW (2008). Abortion and long-term mental health outcomes: a systematic review of the evidence. Contraception.

[29] Thorp JM, Hartmann KE, Shadigian E (2003). Long-term physical and psychological health consequences of induced abortion: review of the evidence. Obstet Gynecol Surv.

[30] Lok IH, Yip AS, Lee DT, Sahota D, Chung TK (2010). A 1-year longitudinal study of psychological morbidity after miscarriage. Fertil Steril.

[31] American Psychiatric Association (2013). DSM-5: Diagnostic and Statistical Manual of Mental Disorders.

