# Supplementary material for: Depressive disorder and grief following spontaneous abortion
Source: BMC Psychiatry. 2016 Apr 12;16:100. doi: 10.1186/s12888-016-0812-y (PMC4830021; doi:10.1186/s12888-016-0812-y)
Supplement: Additional file 1: Table S1. — Type of support preferred by the females after spontaneous abortion. (DOCX 15 kb) [file 12888_2016_812_MOESM1_ESM.docx]

**Additional file 1: Table S1: Type of support preferred by the females after spontaneous abortion**

|  | **Number (%)** |
| --- | --- |
| Talk with a doctor | 70 (51.1) |
| Talk with a nurse, midwife or mental health professional | 23 (16.8) |
| Talk with friend or relative | 2 (1.4) |
| Benefit by referral to a mental health service | 79 (57.7) |
